# Capsaicin Cough Sensitivity and the Association with Clinical Parameters in Bronchiectasis

**DOI:** 10.1371/journal.pone.0113057

**Published:** 2014-11-19

**Authors:** Wei-jie Guan, Yong-hua Gao, Gang Xu, Zhi-ya Lin, Yan Tang, Hui-min Li, Zhi-min Lin, Jin-ping Zheng, Rong-chang Chen, Nan-shan Zhong

**Affiliations:** 1 State Key Laboratory of Respiratory Disease, National Clinical Research Center for Respiratory Disease, Guangzhou Institute of Respiratory Disease, First Affiliated Hospital of Guangzhou Medical University, Guangzhou, Guangdong, China; 2 Department of Respiratory and Critical Care Medicine, First Affiliated Hospital of Zhengzhou University, Zhengzhou, Henan, China; 3 Guangzhou First People's Hospital, Guangzhou, Guangdong, China; University of Dundee, United Kingdom

## Abstract

**Background:**

Cough hypersensitivity has been common among respiratory diseases.

**Objective:**

To determine associations of capsaicin cough sensitivity and clinical parameters in adults with clinically stable bronchiectasis.

**Methods:**

We recruited 135 consecutive adult bronchiectasis patients and 22 healthy subjects. History inquiry, sputum culture, spirometry, chest high-resolution computed tomography (HRCT), Leicester Cough Questionnaire scoring, Bronchiectasis Severity Index (BSI) assessment and capsaicin inhalation challenge were performed. Cough sensitivity was measured as the capsaicin concentration eliciting at least 2 (C_2_) and 5 coughs (C_5_).

**Results:**

Despite significant overlap between healthy subjects and bronchiectasis patients, both C_2_ and C_5_ were significantly lower in the latter group (all P<0.01). Lower levels of C_5_ were associated with a longer duration of bronchiectasis symptoms, worse HRCT score, higher 24-hour sputum volume, BSI and sputum purulence score, and sputum culture positive for *P. aeruginosa*. Determinants associated with increased capsaicin cough sensitivity, defined as C_5_ being 62.5 µmol/L or less, encompassed female gender (OR: 3.25, 95%CI: 1.35–7.83, P<0.01), HRCT total score between 7–12 (OR: 2.57, 95%CI: 1.07–6.173, P = 0.04), BSI between 5–8 (OR: 4.05, 95%CI: 1.48–11.06, P<0.01) and 9 or greater (OR: 4.38, 95%CI: 1.48–12.93, P<0.01).

**Conclusion:**

Capsaicin cough sensitivity is heightened in a subgroup of bronchiectasis patients and associated with the disease severity. Gender and disease severity, but not sputum purulence, are independent determinants of heightened capsaicin cough sensitivity. Current testing for cough sensitivity diagnosis may be limited because of overlap with healthy subjects but might provide an objective index for assessment of cough in future clinical trials.

## Introduction

Bronchiectasis is a debilitating disease in which the vicious cycle of airway infection, augmented inflammation and airway destruction is implicated [1], [2]. Airways secretions and normal ciliary beats are crucial to maintain airway clearance in healthy subjects [3], [4]. In bronchiectasis, these mechanisms are dampened by vicious cycles leading to mucus hypersecretion, which triggers coughing for clearance [5]. Furthermore, persistently augmented cough response [6], [7], which has now been recognized as chronic cough hypersensitivity syndrome [8], [9], might be responsible for productive coughing. However, coughing has been associated with poorer quality of life and insomnia leading to distress and anxiety [10]–[13]. Elucidation of the association of cough and bronchiectasis may facilitate management of cough which is associated with improved quality of life.

The precise mechanisms and characteristics of cough remain poorly elucidated in bronchiectasis. Augmented cough reflex, heightened inflammatory markers (i.e. tachykinin, neurokinin) [14], [15] and up-regulation of transient receptor potential vanilloid subfamily member 1 (TRPV1) could have accounted for chronic cough [14], [16]. However, these measurements have been limited by the invasiveness of sampling (i.e. bronchoscopy) or the lack of well-established diagnostic significance. Clinically, cough reflex sensitivity could be assessed by cough symptom scores, cough frequency and capsaicin inhalation challenge. Because of high feasibility and validity, capsaicin inhalation challenge [6], [7] has become a pivotal objective tool to identify cough hypersensitivity [17]. Characteristics of cough sensitivity in bronchiectasis have been thus far investigated in a single study. Torrego and colleagues [5] documented an increased sensitivity to capsaicin, as evidenced by lower levels of C_5_, which in a linear model, correlated with Leicester Cough Questionnaire (LCQ) scores and FEV_1_. However, the relatively small sample size and lack of delineation of determinants of increased cough sensitivity have rendered the milieu of cough less clear in bronchiectasis. Further elucidation of the characteristics of cough in stable bronchiectasis may provide new insights into future management of cough.

We hypothesized that higher capsaicin cough responsiveness was associated with increased disease severity of bronchiectasis, in terms of more frequent bronchiectasis exacerbations, higher sputum purulence score, presence of cystic bronchiectasis, sputum culture positive for *Pseudomonas aeruginosa* (*P. aeruginosa*) and lower FEV_1_.

We sought to: 1) compare capsaicin cough responsiveness between bronchiectasis patients and healthy subjects; 2) determine the associations between capsaicin cough sensitivity and clinical parameters in terms of radiology, spirometry and sputum bacteriology; 3) explore the determinants of augmented capsaicin cough sensitivity.

## Methods

### Patients

Between September 2012 and November 2013, we performed a screening procedure in The First Affiliated Hospital of Guangzhou Medical University. Diagnosis of bronchiectasis was based on typical clinical symptoms (chronic coughing, sputum production, and/or hemoptysis), and chest high-resolution computed tomography (HRCT) within 12 months of recruitment at the out-patient respiratory clinics. Consecutive patients 18–75 years of age had to remain clinically stable for 4 weeks or more (no significant change in cough frequency or 24-hour sputum volume, no emerging fever, dyspnea or chest pain) [18]. We excluded patients with malignant tumor and those who had used antibiotics, antihistamine or oral corticosteroid, or upper respiratory tract infection within 4 weeks, or limited understanding. In patients with bronchiectasis, both never- and ex-smokers had no active contact with smoking for more than 1 year.

Healthy subjects were 18 to 75 years of age, free of respiratory or systemic diseases, had no upper respiratory tract infection for 4 weeks, and had normal chest radiograph and spirometry. The smoking status is displayed in Table 1.

**Table 1:** Baseline levels.

	Parameter	Bronchiectasis (n = 135)	Healthy subjects (n = 22)	P value
**Demographics**	**Age (yrs)**	44.7±13.7	38.1±13.2	**0.04**
	**Height (cm)**	160.0 (10.0)	163.9±9.7	0.15
	**Weight (kg)**	52.0 (11.0)	62.8±10.5	**<0.01**
	**BMI (kg/m^2^)**	20.0 (4.0)	23.4±3.7	**<0.01**
	**Females (No., %)**	83 (61.5%)	9 (40.9%)	0.07
	**Never-smokers (No., %)**	118 (87.4%)	15 (68.2%)	**0.02**
**Disease-related characteristics**	**Duration of bronchiectasis (yrs)**	10.0 (16.0)	-	-
	**No. of exacerbations within 2 years**	3.0 (3.0)	-	-
	**No. of bronchiectatic lobes**	4.0 (2.0)	-	-
	**HRCT total score**	7.0 (5.0)	-	-
	**24-hour sputum volume (ml)**	20.0 (25.0)	-	-
	**BSI**	6.0 (7.0)	-	-
**Spirometry**	**FVC pred%**	81.8 (26.0)	99.3±11.7	**<0.01**
	**FEV_1_ pred%**	70.8±23.2	99.9±11.9	**<0.01**
	**FEV_1_/FVC (%)**	75.7 (15.7)	84.0±6.4	**<0.01**
	**MMEF pred%**	57.4±30.5	99.8±23.7	**<0.01**
**Bronchiectasis etiology**	**Post-infectious (No., %)**	37 (27.4%)	-	-
	**Immunodeficiency (No., %)**	13 (9.5%)	-	-
	**Gastroesophageal reflux (No., %)**	6 (4.4%)	-	-
	**Other known etiologies** * **(No., %)**	23 (17.0%)	-	-
	**Idiopathic (No., %)**	61 (45.2%)	-	-
**Sputum bacteriology**	***Pseudomonas aeruginosa*** ** (No., %)**	43 (31.9%)	-	-
	**Other PPMs** ** **(No., %)**	38 (28.1%)	-	-
	**Commensals (No., %)**	54 (40.0%)	-	-
**Concomitant medications**	**Mucolytics (No., %)**	103 (76.3%)	-	-
	**Xanthenes (No., %)**	82 (60.7%)	-	-
	**Inhaled corticosteroids (No., %)**	26 (19.3%)	-	-
	**Long-term macrolides (No., %)**	36 (26.7%)	-	-

Numerical data were presented as mean ± standard deviation for normal distribution or otherwise median (interquartile range). Categorical data were expressed as number (percentage) and compared with chi-square test.

PPM: potentially pathogenic microorganism

* Other known etiologies encompassed asthma (n = 7, 5.2%), allergic bronchopulmonary aspergillosis (n = 2, 1.5%), Katargener's syndrome (n = 2, 1.5%), rheumatoid arthritis (n = 2, 1.5%), lung maldevelopement (n = 2, 1.5%), COPD (n = 1, 0.7%), Young's syndrome (n = 1, 0.7%), lung sequestration (n = 1, 0.7%), yellow nail syndrome (n = 1, 0.7%), aspergilloma (n = 1, 0.7%) and eosinophilic bronchiolitis (n = 1, 0.7%). The total number of these counts slightly exceeded that of the table because a minority of patients had dual etiologies.

** Other pathogenic bacteria included *Hemophilus influenzae* (n = 12, 8.9%), *Hemoohilus parainfluenzae* (n = 13, 9.6%), *Staphylococcus aureus* (n = 3, 2.2%), *Klebsiella pneumonae* (n = 3, 2.2%), *Stenotrophic maltophilia* (n = 2, 1.5%), *Escherichia colitis* (n = 1, 0.7%), *Klebsiella ozaenae *(n = 1, 0.7%), *Alcaligenes faecalis subsp faecalis *(n = 1, 0.7%), *Psedumonas pseudoalcaligenes *(n = 1, 0.7%) and *Serratia marcescens* (n = 1, 0.7%).

Data in boldface indicated statistical significance.

No patient was on long-term oral or inhaled antibiotics.

The study protocol was approved by Ethics Committee of First Affiliated Hospital of Guangzhou Medical University. All subjects gave written informed consent.

### Study design

This was a single center, cross-sectional study. For bronchiectasis patients, meticulous history taking, Leicester cough Questionnaire (LCQ) [19], [20] and cough symptom scoring, capsaicin cough provocation test, spirometry, spontaneous or induced sputum sampling, and sputum bacterial culture were performed. Healthy subjects underwent capsaicin cough provocation test and spirometry only.

### Capsaicin inhalation challenge

The methodology of capsaicin provocation test, by using dosimeter approach, has been introduced previously [5]. Briefly, subjects were instructed not to suppress cough or speak during the measurement. Capsaicin dilutions with doubling concentrations (1.95 µmol∼1000 µmol) were delivered via Masterscreen (Output: 160 µl/min) and an automated APSpro system (Jaeger, Carefusion Co. Ltd, Hochberg, Germany). Subjects were not aware of the capsaicin concentrations throughout the measurement. Inhalation challenge using natural saline served as blank control. Repetitive inhalation challenges were performed at 1-min intervals, during which the number of coughs within the first 30 s was recorded. Inhalation challenge was ceased in case of 5 or more coughs, the corresponding capsaicin concentration (C_5_) was recorded. Similarly, the C_2_ was also documented. If less than 2 or 5 coughs were produced at the final step, 1000 µmol/L was artificially assigned for C_2_ or C_5_, respectively.

In this study, cough hypersensitivity denoted the C_5_ being 62.5 µmol/L or less.

### Spirometry

Spirometry was conducted by using spirometers (QUARK PFT, COSMED Co. Ltd., Milan, Italy) in accordance with the *American Thoracic Society*/*European Respiratory Society* guideline [21]. Results were derived from at least 3 maneuvers, with between-maneuver variation of <5% or 200 ml in FVC and FEV_1_. Maximal FVC and FEV_1_ were reported. Predicted values were recommended by Zheng and associates [22].

### Sputum sampling

The 24-hour sputum was assessed in 3 consecutive days. Patients were, following removal of contents in oral cavity, instructed to expectorate in 50 ml sterile transparent containers which were conveyed to the designated research nurse at the hospital visit. 24-hour sputum volume was trichotomised into 0–10 ml, 11–30 ml and >30 ml, respectively.

Fresh sputum was sampled during the hospital visit for bacterial culture. For subjects with insufficient sputum for analysis, induction using graded concentrations of hypertonic saline (3%, 5% and 7% when appropriate) was performed. Sputum induction for bacterial culture has been validated previously [23]. Qualified sputum sample, defined as leukocyte count >25 and squamous epithelial cell <10 per vision (magnified ×100), was selected. This entailed rapid transmission to microbiology laboratory (within 2 hours) for culture.

Sputum purulence as scored for 1, 2, 3, 4, 5, 6, 7 and 8 points corresponding to complete absence, almost translucent, half translucent, translucent but colorless, opaque and white, grey and green, moderately green and dark green, respectively [18].

### Sputum bacterial culture

Please refer to File S1.

### Cough symptom score

The severity of daytime and nighttime cough symptoms was scored by patients [5]. Daytime and night time scores were pooled resulting in total cough symptom score, with a maximum of 10 points. (see Table S1 in File S1)

### Clinical assessments

The methodologies of bronchiectasis etiology assessments have been described previously [24]–[26], with slight modifications. Post-infectious bronchiectasis referred to the bronchiectasis symptoms that developed after any infectious diseases, including measles, tuberculosis, severe pneumonia, pertussis and other clinically significant diseases. Idiopathic bronchiectasis denoted the exclusion of any known causes.

The HRCT score was calculated on a lobar basis by using modified Reiff score, with lingular being a separate lobe, to assess the morphology of bronchiectasis (0 for nil, 1 for tubular, 2 for varicose and 3 for cystic bronchiectasis, respectively) [24]. The maximal possible score was 18.

Bronchiectasis Severity Index (BSI) [27], a novel tool previously validated, was applied to determine the severity of bronchiectasis. The BSI was a composite of clinical parameters, including the age, body-mass index, prior exacerbations and prior hospitalization in the preceding year, Medical Research Council dyspnea score, FEV_1_ predicted%, *P. aeruginosa* infection, colonization with other potentially pathogenic microorganisms (PPMs) and the number of bronchiectatic lobes. The cutoff values of BSI were ≤4, 5–8 and ≥9 for mild, moderate and severe bronchiectasis, respectively [27].

The LCQ, validated for use both in English and Chinese patients with bronchiectasis in previous studies [19], [20] and the study by our research group (Gao YH, et al. Validation of Chinese Leicester Cough Questionnaire in patients with bronchiectasis, article awaiting for publication in *International Journal of Tuberculosis and Lung Disease*), was a measure of quality of life. The LCQ consisted of 19 items divided in 3 domains, with a lower score indicating more significant impacts of cough on daily life. The lowest and highest possible total score was 0 and 21, respectively.

### Statistical analysis

Statistical analysis was performed using SPSS 16.0 version package (SPSS Inc., Chicago, IL, USA) and Graphpad Prism 5.0 (Graphpad Inc., San Diego, USA. Numerical data were presented as mean ± standard deviation for normal distribution or otherwise median (interquartile range). Independent t-test or Mann-Whitney test was adopted for two-group comparisons as appropriate. Three-group comparisons were made using one-way analysis of variance or Kruskal-Wallis test as indicated. Categorical data were expressed as number (percentage) and compared with chi-square test. The correlation analysis was done using Spearman's model. The data of C_2_ and C_5_ were subjected to logarithmic transformation (with the base of 10) for further analyses. Determinants of increased cough sensitivity were identified using univariate Logistic regression analysis, data with P value of 0.10 or less were entered into multivariate regression model using backward selection algorithm. P<0.05 was deemed statistical significant for all analyses.

## Results

### Subject recruitment

See Figure 1.

**Figure 1:**
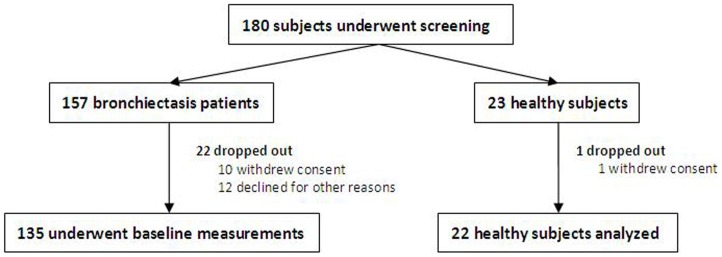
Subject recruitment flowchart. We screened 180 subjects (157 bronchiectasis patients and 23 healthy subjects). Of these, 22 bronchiectasis patients and 1 healthy subjects dropped out, and finally the data of 135 bronchiectasis patients and 22 healthy subjects were analyzed.

### Baseline levels

Compared with healthy subjects, bronchiectasis patients had a higher mean age, and lower weight and BMI (all P<0.05). Markedly reduced spirometry was found in bronchiectasis patients (all P<0.01). Overall, our cohort represented patients with predominantly mild-to-moderate bronchiectasis (median BSI: 7.0). In terms of sputum bacteriology, commensals accounted for 40.0%, and the most common PPM was *P. aeruginosa* (31.9%, of whom 28.1% had chronic infection). The most common etiology was idiopathic (45.2%), followed by post-infectious (27.4%). Mucolytics (76.3%) and theophylline (60.7%) were the most frequently used concomitant medications. (Table 1)

### Capsaicin cough sensitivity in bronchiectasis patients vs. healthy subjects

**Figure 2:**
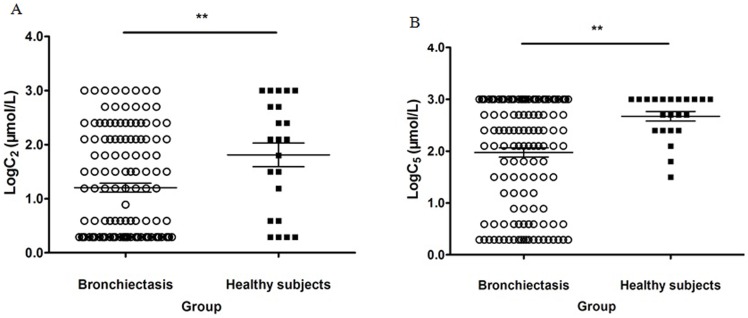
Distribution of Log_10_C_2_ and Log_10_C_5_ in bronchiectasis patients and healthy subjects. The capsaicin cough sensitivity, calculated as the capsaicin concentration causing at least 2 (C_2_) and 5 (C_5_), was compared between bronchiectasis patients and healthy subjects. Bronchiectasis patients yielded markedly lower levels of C_2_ and C_5_, suggesting significantly higher capsaicin cough sensitivity than healthy subjects. The lines of the bar in each figure represented the 25% percentile, median and 75% percentile, respectively. Figure 2-A, distribution of Log_10_C_2_, Figure 2-B, Distribution of Log_10_C_5_. The asterisks in both Figures 2-A and 2-B denoted P<0.01.

There was an overlap in capsacin cough sensitivity between healthy subjects and bronchiectasis patients. However, logarithms of C_2_ and C_5_ were significantly lower in bronchiectasis patients (all *P*<0.01), suggesting a higher magnitude of capsaicin cough sensitivity than healthy subjects. (Figures 2-A and 2-B)

### Capsaicin cough sensitivity of bronchiectasis patients when stratified by sex and disease duration

Compared with males, significantly lower levels of C_2_ and C_5_ (both *P*<0.01) were observed in female patients. However, the C_2_ and C_5_ were not associated with the disease duration. (Table 2)

**Table 2:** Comparison on capsaicin cough sensitivity, cough symptoms and quality of life in bronchiectasis.

	Duration of bronchiectasis symptoms (yrs)				HRCT total score				Sex		
Parameter	<5	5–10	>10	P	1–6	7–13	14–18	P	Males	Females	P
**Log_10_C_2_ (µmol)**	0.59 (1.81)	0.59 (1.51)	1.19 (2.03)	0.42	1.19 (2.03)	0.59 (1.81)	0.59 (1.81)	0.83	1.65 (2.11)	0.59 (1.36)	**<0.01**
**Log_10_C_5_ (µmol)**	2.70 (1.20)	1.95 (2.41)	2.40 (2.03)	0.20	2.70 (0.90)	1.80 (2.41)	1.60±0.97	**0.01**	2.70 (0.90)	2.10 (2.26)	**<0.01**
**Cough symptom score**	2.00 (2.00)	4.00 (2.00)	3.0 (3.00)	**<0.01**	2.50 (2.00)	3.00 (2.00)	4.87±2.10	**<0.01**	3.00 (2.00)	3.0 (2.00)	0.31
**LCQ total score**	14.80±3.60	11.71±3.28	12.09±4.17	**<0.01**	13.74±3.78	11.90±3.76	10.52±3.24	**<0.01**	12.76±3.53	12.56±4.08	0.73

	Sputum purulence score				24-hour sputum volume (ml)				FEV1 predicted		
	≤2	3–5	6–7	P	≤10	11–30	>30	P	>50%	≤50%	P
**Log_10_C_2_ (µmol)**	0.59 (2.26)	1.65 (2.11)	0.59 (1.81)	0.11	0.59 (1.81)	0.59 (1.73)	1.00 (2.11)	0.59	1.19 (1.81)	0.59 (1.88)	0.91
**Log_10_C_5_ (µmol)**	2.70 (1.95)	3.00 (0.97)	2.93 (2.33)	**<0.01**	2.55 (1.50)	2.40 (2.11)	1.50 (1.81)	**0.04**	2.40 (1.81)	1.69±1.04	0.15
**Cough symptom score**	2.08±0.49	2.52±0.30	3.00 (2.00)	**<0.01**	2.00 (2.00)	3.00 (2.00)	4.00 (2.00)	**<0.01**	3.00 (2.00)	3.50 (3.00)	0.07
**LCQ total score**	12.43±1.40	14.83±0.77	12.01±0.35	**<0.01**	14.06±4.10	12.42±3.16	10.60±3.54	**<0.01**	12.86±3.81	11.74±4.05	0.17

	BSI				Sputum bacteriology				Cystic bronchiectasis		
	Mild (0–4)	Moderate (5–8)	Severe (≥9)	P	Commensals	Other PPMs	*P. aeruginosa*	P	Nil	Yes	P
**Log_10_C_2_ (µmol)**	1.50 (2.11)	0.44 (1.51)	0.59 (1.66)	**0.03**	1.19 (2.11)	0.92 (2.03)	0.44 (0.98)	**0.02**	0.59 (1.88)	0.59 (1.81)	0.80
**Log_10_C_2_ (µmol)**	2.70 (0.90)	2.10 (2.41)	1.50 (1.81)	**<0.01**	2.70 (1.66)	2.40 (1.30)	1.19 (1.88)	**<0.01**	2.70 (2.41)	2.10 (1.81)	0.13
**Cough symptom score**	2.00 (2.00)	3.00 (2.00)	4.00 (2.00)	**<0.01**	3.00 (2.00)	3.00 (2.00)	4.00 (2.00)	**0.01**	3.00 (2.00)	3.00 (3.00)	**<0.01**
**LCQ total score**	13.46±4.03	12.83±2.26	11.18±4.08	**0.02**	13.57±4.26	12.61±3.56	11.48±3.33	**0.03**	13.18±3.89	12.21±3.81	0.16

Numerical data were presented as mean ± standard deviation for normal distribution or otherwise median (interquartile range). Categorical data were expressed as number (percentage) and compared with chi-square test.

PPM: potentially pathogenic microorganism.

Data in boldface indicated statistical significance.

### Capsaicin cough sensitivity and disease severity of bronchiectasis

Higher BSI and sputum culture positive for *P. aeruginosa* were associated with significantly lower levels of C_2_ and C_5_ (both *P*<0.05). Lower levels of C_5_, but not C_2_, were observed in patients with worse HRCT total score, increased sputum purulence score and higher 24-hour sputum volume (all *P*<0.05). However, FEV_1_ being 50% predicted or less and presence of cystic bronchiectasis did not affect capsaicin cough sensitivity. (Table 2)

### Capsaicin cough sensitivity and Quality of life (QoL) indicators

Overall, capsaicin cough sensitivity yielded differences from QoL measures in their capacity of reflecting the disease severity. Higher capsaicin cough sensitivity (lower C_2_ and C_5_) and poorer QoL (higher cough symptom score and/or lower LCQ total score) were consistently associated with higher BSI and sputum culture positive for *P. aeruginosa* (both *P*<0.05). The C_5_, but not C_2_, was a marker more closely correlated with cough symptom score and LCQ total score. Neither capsaicin cough sensitivity nor QoL measures could effectively differentiate patients with lower FEV_1_. (Table 2)

### Bronchiectasis with and without capsaicin cough hypersensitivity

Bronchiectasis patients with capsaicin cough hypersensitivity were associated with markedly lower body weight, BMI, FVCpred%, FEV_1_pred%, MMEFpred%, LCQ total score and the proportion of other PPMs, than their counterparts (all P<0.05). Furthermore, significantly higher proportion of females, higher sputum purulence score, worse HRCT total score, BSI, cough symptom score and sputum culture positive for *P. aeruginosa* were observed in patients with augmented capsaicin cough sensitivity (all P<0.05). The differences in the age, height, duration of bronchiectasis, 24-hour sputum volume, FEV_1_/FVC ratio, commensals present in sputum and the categories of bronchiectasis etiologies were unremarkable (all *P*>0.05). (Table 3)

**Table 3:** Comparison on clinical characteristics of bronchiectasis patients with and without capsaicin cough hypersensitivity.

	Parameter	Cough hypersensitivity (n = 52)	Normal cough sensitivity (n = 83)	P value
**Demographics**	**Age (yrs)**	45.4±15.3	44.2±12.7	0.62
	**Height (cm)**	160.0±7.2	162.2±7.6	0.09
	**Weight (kg)**	49.0 (8.2)	54.0 (11.5)	**<0.01**
	**BMI (kg/m^2^)**	19.4±2.6	21.1±3.2	**<0.01**
	**Females (No., %)**	41 (78.8%)	42 (50.6%)	**<0.01**
**Disease-related characteristics**	**Duration of bronchiectasis (yrs)**	10.0 (15.0)	10.0 (17.0)	0.70
	**No. of bronchiectatic lobes**	4.0 (3.0)	3.0 (3.0)	**<0.01**
	**HRCT total score**	8.0 (6.0)	5.0 (6.0)	**<0.01**
	**24-hour sputum volume (ml)**	20.0 (45.0)	15.0 (20.0)	0.07
	**BSI**	7.4±3.5	5.0 (6.0)	**<0.01**
	**Sputum purulence score**	6.0 (1.0)	6.0 (2.0)	**0.02**
**Spirometry**	**FVC pred%**	71.9±19.8	83.3±18.6	**<0.01**
	**FEV_1_ pred%**	62.7±22.0	75.9±22.7	**<0.01**
	**FEV_1_/FVC (%)**	71.4±13.2	76.8 (15.2)	0.12
	**MMEF pred%**	48.6±27.8	63.0±31.0	**<0.01**
**Cough-related parameters**	**Cough symptom score**	4.0 (2.0)	3.0 (2.0)	**<0.01**
	**LCQ total score**	11.7±3.9	13.2±3.7	**0.03**
**Bronchiectasis etiology**	**Post-infectious (No., %)**	14 (26.9%)	23 (27.7%)	0.58
	**Immunodeficiency (No., %)**	7 (13.5%)	6 (7.2%)	0.23
	**Gastroesophageal reflux (No., %)**	2 (3.8%)	4 (4.8%)	0.87
	**Other known etiologies (No., %)**	7 (13.5%)	15 (18.1%)	0.48
	**Idiopathic (No., %)**	24 (46.2%)	27 (32.5%)	0.11
**Sputum bacteriology**	***Pseudomonas aeruginosa*** ** (No., %)**	27 (51.9%)	16 (19.3%)	**<0.01**
	**Other PPMs (No., %)**	9 (17.3%)	29 (34.9%)	**0.03**
	**Commensals (No., %)**	16 (30.8%)	38 (45.8%)	0.08

Numerical data were presented as mean ± standard deviation for normal distribution or otherwise median (interquartile range). Categorical data were expressed as number (percentage) and compared with chi-square test.

PPM: potentially pathogenic microorganism.

A minority of patients had dual etiologies, therefore the sum of proportion of individual etiologies was greater than 100%.

Data in boldface indicated statistical significance.

### Univariate and multivariate analyses of capsaicin cough hypersensitivity

In univariate analysis, being females, the duration of cough between 5 and 10 years, HRCT total score being 7 or greater, BSI being 5 or greater, 24-hour sputum volume between 11 and 29 ml, cough symptom total score greater than 5 and sputum culture positive for *P. aeruginosa* (all P<0.05) were independently associated with capsaicin cough hypersensitivity, defined as the C_5_ being 62.5 µmol or less. (see Table S2 in File S1)

In multivariate analysis, after adjusting for potential confounders, the final determinants encompassed female gender (OR: 3.25, 95%CI: 1.35–7.83, P<0.01), HRCT total score between 7 and 12 (OR: 2.57, 95%CI: 1.07–6.17, P = 0.04), BSI between 5 and 8 (OR: 4.05, 95%CI: 1.48–11.06, P<0.01) and 9 or greater (OR: 4.38, 95%CI: 1.48–12.93, P<0.01). (Table 4)

**Table 4:** Multivariate analysis of the determinants of capsaicin cough hypersensitivity.

Variables	C5≤62.5 µmol (No., %)	C5>62.5 µmol (No., %)	Multivariable regression model		
			OR	95%CI	P
**Sex**					
** Male**	11 (21.2)	41 (49.4%)	-	Reference	-
** Female**	**41 (78.8%)**	**42 (50.6%)**	**3.25**	**1.35-7.83**	**<0.01**
**HRCT total score**					
** ≤6**	16 (30.8%)	50 (60.2%)	-	Reference	-
** 7-12**	**28 (53.8%)**	**26 (31.3%)**	**2.57**	**1.07-6.17**	**0.04**
** ≥13**	**8 (15.4%)**	**7 (8.4%)**	**2.32**	**0.66-8.17**	**0.19**
**BSI**					
** 0-4**	11 (21.2%)	41 (49.4%)	-	Reference	-
** 5-8**	24 (46.2%)	25 (30.1%)	4.05	1.48-11.06	<0.01
** ≥9**	**17 (32.7%)**	**17 (20.5%)**	**4.38**	**1.48-12.93**	**<0.01**

Categorical data were expressed as number (percentage) and compared with chi-square test.

Data in boldface indicated statistical significance.

In multivariate analysis, settings of the covariates were as follows: Sex: 0 for female, 1 for males; duration of cough: 0 for less than 5 years, 1 for 5 to 10 years, 2 for greater than 10 years; HRCT score: 0 for 6 or less, 1 for greater than 7 and less than 13, 2 for 13 or greater; bronchiectasis severity index: 0 for 4 or less, 1 for 5 to 8, 2 for 9 or greater; 24-hour sputum volume: 0 for 10 ml or less, 1 for greater than 10 and less than 30 ml, 2 for 30 ml or greater; cough symptom score: 0 for 5 or less, 1 for greater than 5; sputum bacteriology: 0 for commensals, 1 for *Pseudomonas aeruginosa*, 2 for other PPMs.

## Discussion

### Principal findings

Our data demonstrated that C_2_ and C_5_ were significantly lower in bronchiectasis patients, despite that healthy subjects and bronchiectasis patients harbored a notable overlap in capsaicin cough sensitivity. Lower levels of C_5_ were associated with a longer duration of bronchiectasis symptoms, worse HRCT score, 24-hour sputum volume and BSI, higher sputum purulence score and sputum culture positive for *P. aeruginosa*. Patients with increased cough sensitivity had poorer clinical conditions, including higher BSI and HRCT score, than their counterparts. Determinants associated with increased capsaicin cough sensitivity consisted of female gender, HRCT score between 7 and 12, and BSI of 5 or greater.

### Interpretations

The current study partially contradicted the findings by Torrego et al [5], which showed no association between cough sensitivity and the severity of bronchiectasis, the latter of which was referred to as FEV_1_ predicted and LCQ scores. This disparity could be interpreted by the different tools and methodologies employed. Indeed, the disease severity should be better assessed by using the age, gender, smoking history, sputum culture positive for *P. aeruginosa*, spirometry, radiological findings and the underlying etiology [28], [29] The BSI is a novel tool to determine future risks of hospitalizations, exacerbations, quality of life and mortality [27], which comprehensively comprised different sets of clinical parameters. Furthermore, our systematic analyses based on stratification of multiple parameters, including chest HRCT characteristics, sputum bacteriology and the previous history, might have increased the likelihood of unraveling these associations. The fact that patients with cough hypersensitivity were characterized by increased disease severity also reaffirmed our findings.

A notable clinical significance of our study was that capsaicin cough sensitivity was an indirect reflection of the disease severity of bronchiectasis and influenced by a battery of clinical parameters. Although the capsaicin cough sensitivity was not an ideal parameter for discrimination of bronchiectasis from healthy subjects, or identifying certain subgroups of bronchiectasis patients, it might be promising for the prospective follow-up and assessment of the therapeutic outcomes of cough. For instance, the efficacy of atorvastatin on bronchiectasis has recently been proven by using LCQ scores as a primary endpoint [30]. Our study warrants the use of capsaicin cough sensitivity as an objective parameter for assessment of cough in future clinical trials. Furthermore, early management of bronchiectasis via regular pharmacological intervention (i.e. mucolytics and bronchodilators for airway clearance, antibiotics for suppressing *P. aeruginosa* growth) may be associated with reduced likelihood of having persistent severe cough and disrupted daily life. Understanding of the mechanisms and characteristics of cough in bronchiectasis is expected to facilitate the development of such medications.

The exact mechanisms underlying varying responses to capsaicin challenge remain unknown, but could be related to the different magnitude of airway inflammation, airway destruction, characters of coughing (dry vs. wet cough), psychological status and inter-individual variability. Furthermore, the heterogeneous underlying causes of bronchiectasis might have contributed significantly to the observed differences in capsaicin cough sensitivity.

More importantly, we have disclosed that determinants associated with increased capsaicin cough sensitivity encompassed female gender, HRCT score between 7 and 12, and BSI of 5 or greater. Although our cohort was predominated by females, we found that a greater proportion of females harbored the *bona fide* cough hypersensitivity, which was in concert with literature reports [31]. The worse HRCT total score and higher BSI have been associated with cough hypersensitivity, further reaffirming that cough sensitivity mirrored the disease severity of bronchiectasis.

The most interesting finding was that the 24-hour sputum volume, FEV_1_ predicted% and cystic bronchiectasis did not accurately predict cough sensitivity. A possible interpretation could be that capsaicin cough sensitivity was solely related to upper airway sensory nerve (esp. C-fibers) sensitivity. Or it could be that miscellaneous clinical parameters have masked their underlying effects. Despite that sputum purulence or color has been suggested as a better indicator of cough sensitivity, since these parameters have been associated with the severity of inflammation, airway destruction and proteolytic activity [32], our data failed to confirm this finding. Moreover, although antitussives are not recommended for suppressing cough in bronchiectasis, treatments specifically targeting at alleviating severe cough and antagonizing cough-related inflammatory mediators may be promising alternatives. However, it should be recognized that our definition of cough hypersensitivity with C_5_ alone might have biased the findings. However, before miscellaneous endpoints are further validated, the C_5_ remains an optimal parameter for assessments.

### Strengths and limitations

Strengths of our study comprised the relatively large sample size, systematic comparison of cough sensitivity stratified by various clinical parameters, delineation of the characteristics of patients with different cough sensitivities and the identification of determinants of cough hypersensitivity.

Some demerits must be underlined. First, this was not large-scale prospective study investigating the utility of capsaicin cough sensitivity to predict the prognosis of bronchiectasis. Second, our healthy subject cohort and bronchiectasis patients were not matched for age and sex. Third, cough symptoms and severity lacked gold standards, i.e. 24-hour ambulatory cough monitoring. However, such measurements would be less cost-effective for screening. Fourth, it would be more ideal to apply capsaicin cough challenge test in individual or prospective studies. Fifth, miscellaneous inflammatory biomarkers (i.e. neutrophil elastase and matrix metalloproteinase) [33] were not assayed in the current study. However, since this was not a mechanistic study, we were unable to extrapolate to the mechanisms underlying patients with cough hypersensitivity. Furthermore, the imbalanced proportion of never-smokers in healthy subjects and bronchiectasis patients might help interpret the significant overlap in capsaicin cough sensitivity. Finally, our dosimeter used to administer capsaicin was not limited for constant inspiratory flow.

### Conclusion

We have further validated capsaicin cough challenge test in bronchiectasis. Capsaicin cough sensitivity is heightened in a subgroup of bronchiectasis patients and associated with the disease severity. Gender and disease severity are independent determinants. Despite that current testing for cough sensitivity may be limited because of the overlap with healthy subjects, our findings may provoke further studies that might improve quality of life via amelioration of cough hypersensitivity. Capsaicin challenge test has been added as a tool in bronchiectasis and has potential use in future studies to assess objective improvement in cough hypersensitivity.

## Supporting Information

File S1 **Supporting Information.** Table S1, Cough symptom scores. Table S2, Univariate analysis of the determinants of capsaicin cough hypersensitivity. Categorical data were expressed as number (percentage) and compared with chi-square test. Data in boldface indicated statistical significance. In multivariate analysis, settings of the covariates were as follows. Sex: 0 for female, 1 for males; duration of cough: 0 for less than 5 years, 1 for 5 to 10 years, 2 for greater than 10 years; HRCT score: 0 for 6 or less, 1 for greater than 7 and less than 13, 2 for 13 or greater; bronchiectasis severity index: 0 for 4 or less, 1 for 5 to 8, 2 for 9 or greater; 24-hour sputum volume: 0 for 10 ml or less, 1 for greater than 10 and less than 30 ml, 2 for 30 ml or greater; cough symptom score: 0 for 5 or less, 1 for greater than 5; sputum bacteriology: 0 for commensals, 1 for *Pseudomonas aeruginosa*, 2 for other PPMs.
(DOC)
