# Supplementary material for: Capsaicin Cough Sensitivity and the Association with Clinical Parameters in Bronchiectasis
Source: PLoS One. 2014 Nov 19;9(11):e113057. doi: 10.1371/journal.pone.0113057 (PMC4237391; doi:10.1371/journal.pone.0113057)
Supplement: File S1 — Supporting Information. Table S1, Cough symptom scores. Table S2, Univariate analysis of the determinants of capsaicin cough hypersensitivity. Categorical data were expressed as number (percentage) and compared with chi-square test. Data in boldface indicated statistical significance. In multivariate analysis, settings of the covariates were as follows. Sex: 0 for female, 1 for males; duration of cough: 0 for less than 5 years, 1 for 5 to 10 years, 2 for greater than 10 years; HRCT score: 0 for 6 or less, 1 for greater than 7 and less than 13, 2 for 13 or greater; bronchiectasis severity index: 0 for 4 or less, 1 for 5 to 8, 2 for 9 or greater; 24-hour sputum volume: 0 for 10 ml or less, 1 for greater than 10 and less than 30 ml, 2 for 30 ml or greater; cough symptom score: 0 for 5 or less, 1 for greater than 5; sputum bacteriology: 0 for commensals, 1 for Pseudomonas aeruginosa, 2 for other PPMs. (DOC) [file pone.0113057.s001.doc]

**Original article**

**Capsaicin cough sensitivity and the association with clinical parameters in bronchiectasis**

**Wei-jie Guan ^*1^, Ph.D., Yong-hua Gao ^*2^, Ph.D., Gang Xu ^*3^, Ph.D., Zhi-ya Lin ^1^, Ph.D., Yan Tang ^1^, M.D., Hui-min Li ^1^, M.T., Zhi-min Lin ^1^, MSc., Jin-ping Zheng ^1^, M.D., Rong-chang Chen ^1^, M.D., Nan-shan Zhong ^1^, M.D.**

^1^ State Key Laboratory of Respiratory Disease, National Clinical Research Center for Respiratory Disease, Guangzhou Institute of Respiratory Disease, First Affiliated Hospital of Guangzhou Medical University, Guangzhou, Guangdong, China

^2^ Department of Respiratory and Critical Care Medicine, First Affiliated Hospital of Zhengzhou University, Zhengzhou, Henan, China

^3^ Guangzhou First People’s Hospital, Guangzhou, Guangdong, China

**Corresponding author 1:** Nan-shan Zhong, M. D., State Key Laboratory of Respiratory Disease, National Clinical Research Center for Respiratory Disease, Guangzhou Institute of Respiratory Disease, First Affiliated Hospital of Guangzhou Medical University, Address: 151 Yanjiang Road, Guangzhou, Guangdong, China, Fax: +86-20-83062718, Phone: +86-20-83062718, E-mail: [nanshan@vip.163.com](mailto:nanshan@vip.163.com)

**Corresponding author 2:** Rong-chang Chen, M. D., State Key Laboratory of Respiratory Disease, National Clinical Research Center for Respiratory Disease, Guangzhou Institute of Respiratory Disease, First Affiliated Hospital of Guangzhou Medical University, Address: 151 Yanjiang Road, Guangzhou, Guangdong, China, Fax: +86-20-83062719, Phone: +86-20-83062719, E-mail: chenrc@vip.163.com

***These authors contributed equally to the study.**

**Methods**

**Cough symptom scores**

**Table S1 Cough symptom scores**

| **Scale** | **During the day** | **During the night** |
| --- | --- | --- |
| 0 | No cough | No cough |
| 1 | Cough for one short period | Cough on waking in the morning only |
| 2 | Cough for two or more short periods | Wake once or early due to cough |
| 3 | Frequent coughing, not interfering with usual daytime activities | Frequent waking due to coughs |
| 4 | Frequent coughing, interfering with usual daytime activities | Frequent coughs most of the night |
| 5 | Distressing coughs for most of the day | Distressing coughs preventing any sleep |

**Sputum culture for bacteriologic assessment**

Blood agar (Biomeurix Co. Ltd) and chocolate agar plates (Biomeurix Co. Ltd) were adopted for bacterial culture. Fresh sputum was homogenized using SPUTASOL (Oxoid SR089A) and inoculated with standardized inoculation rings. Culture media were placed in a thermostatic box containing 5% carbon dioxide at 37℃ for overnight inoculation. Pathogenic bacteria included *Pseudomonas aeruginosa*, *Hemophilus influenzae*, *Hemophilus parainfluenzae*, *Streptococcus pneumonae*, *Staphylococcus aureus* and *Moraxella catarrhalis*. Non-pathogenic bacteria (commensals) included *Neisseria*, α-*Streptococcus hemolyticus*, Bacilli *diphtheria* and coagulase-negative *staphylococcus*. Prolonged culture (up to 4 days) was done for negative plates.

**Table S2 Univariate analysis of the determinants of capsaicin cough hypersensitivity**

| **Variables** | **C5 ≤62.5μmol (No., %)** | **C5 >62.5μmol (No., %)** | **Univariable regression model** | | |
| --- | --- | --- | --- | --- | --- |
|  |  |  | **OR** | **95%CI** | **P** |
| **Sex** |  |  |  |  |  |
| **Male** | 11 (21.2) | 41 (49.4%) | - | Reference | - |
| **Female** | **41 (78.8%)** | **42 (50.6%)** | **3.64** | **1.65-8.04** | **<0.01** |
| **Age (yrs)** |  |  |  |  |  |
| **18-35** | 15 (28.8%) | 24 (28.9%) | - | Reference | - |
| **36-49** | 16 (30.8%) | 29 (34.9%) | 0.88 | 0.36-2.15 | 0.78 |
| **≥50** | 21 (40.4%) | 30 (36.1%) | 1.12 | 0.48-2.63 | 0.79 |
| **Duration of cough (yrs)** |  |  |  |  |  |
| **<5** | 9 (17.3%) | 25 (30.1%) | - | Reference | - |
| **5-10** | **23 (44.2%)** | **23 (27.7%)** | **2.78** | **1.07-7.23** | **0.03** |
| **≥10** | 20 (38.5%) | 35 (42.2%) | 1.59 | 0.62-4.06 | 0.33 |
| **HRCT total score** |  |  |  |  |  |
| **≤6** | 16 (30.8%) | 50 (60.2%) | - | Reference | - |
| **7-12** | **28 (53.8%)** | **26 (31.3%)** | **3.37** | **1.55-7.31** | **<0.01** |
| **≥13** | **8 (15.4%)** | **7 (8.4%)** | **3.57** | **1.12-11.40** | **0.03** |
| **BSI** |  |  |  |  |  |
| **0-4** | 11 (21.2%) | 41 (49.4%) | - | Reference | - |
| **5-8** | **24 (46.2%)** | **25 (30.1%)** | **3.58** | **1.50-8.54** | **<0.01** |
| **≥9** | **17 (32.7%)** | **17 (20.5%)** | **3.73** | **1.45-9.60** | **<0.01** |
| **24-hour sputum volume (ml)** |  |  |  |  |  |
| **≤10** | 18 (34.6%) | 35 (42.2%) | - | Reference | - |
| **11-29** | **17 (32.7%)** | **34 (41.0%)** | **0.97** | **0.43-2.19** | **0.95** |
| **≥30** | 17 (32.7%) | 14 (16.8%) | 2.36 | 0.95-5.85 | 0.06 |
| **Cough symptom score** |  |  |  |  |  |
| **≤5** | 43 (82.7%) | 79 (95.2%) | - | Reference | - |
| **>5** | **9 (17.3%)** | **4 (4.8%)** | **4.13** | **1.20-14.22** | **0.02** |
| **FEV_1_ pred%<50%** |  |  |  |  |  |
| **No** | 39 (75.0%) | 70 (84.3%) | - | Reference | - |
| **Yes** | 13 (25.0%) | 13 (15.7%) | 1.80 | 0.76-4.25 | 0.18 |
| **Cystic bronchiectasis** |  |  |  |  |  |
| **Nil** | 21 (40.4%) | 39 (47.0%) | - | Reference | - |
| **Yes** | 31 (59.6%) | 44 (53.0%) | 1.31 | 0.65-2.64 | 0.45 |
| **Sputum bacteriology** |  |  |  |  |  |
| ***P. aeruginosa*** | **27 (51.9%)** | **16 (19.3%)** | **4.01** | **1.71-9.39** | **<0.01** |
| **Other PPMs** | 9 (17.3%) | 29 (34.9%) | 0.74 | 0.29-1.90 | 0.53 |
| **Commensals** | 16 (30.8%) | 38 (45.8%) | - | Reference | - |

Categorical data were expressed as number (percentage) and compared with chi-square test.

Data in boldface indicated statistical significance

In multivariate analysis, settings of the covariates were as follows. Sex: 0 for female, 1 for males; duration of cough: 0 for less than 5 years, 1 for 5 to 10 years, 2 for greater than 10 years; HRCT score: 0 for 6 or less, 1 for greater than 7 and less than 13, 2 for 13 or greater; bronchiectasis severity index: 0 for 4 or less, 1 for 5 to 8, 2 for 9 or greater; 24-hour sputum volume: 0 for 10ml or less, 1 for greater than 10 and less than 30ml, 2 for 30 ml or greater; cough symptom score: 0 for 5 or less, 1 for greater than 5; sputum bacteriology: 0 for commensals, 1 for *Pseudomonas aeruginosa*, 2 for other PPMs.
